# Advancing Minimally Invasive Cardiac Surgery—Let’s Take a Look into the Future

**DOI:** 10.3390/jcm14030904

**Published:** 2025-01-29

**Authors:** Manuel Wilbring

**Affiliations:** Center for Minimally Invasive Cardiac Surgery, University Heart Center Dresden, 01328 Dresden, Germany; manuel.wilbring@gmail.com; Tel.: +49-351-450-1606

The evolution of cardiac surgery over the last two decades has been nothing short of revolutionary. Minimally invasive cardiac surgery (MICS) has emerged as a transformative approach, offering patients reduced surgical trauma, faster recovery times, and improved outcomes. Since the pioneering work in minimally invasive cardiac surgery of Rao and Kumar, as well as Sabik and Cosgrove, a continuous process of advancement was set in motion [1,2,3,4,5,6,7,8,9]. Moreover, the emergence of catheter-based procedures further accelerated the evolution of minimally invasive surgical techniques [10,11,12].

As cardiac surgeons, we stand at the precipice of a significant paradigm shift. The conventional wisdom that full sternotomy is the gold standard for valve surgery is being increasingly challenged by compelling data supporting MICS. For example, the extensive results from the Dresden group’s landmark study on 1000 consecutive patients undergoing transaxillary minimally invasive aortic valve replacement underscore this point [12]. The findings reveal not only the safety and efficacy of this approach but also its potential to become the dominant access route for a wide array of cardiac procedures.

A striking aspect of this transaxillary MICS series is the dramatic increase in its utilization over the study period, rising from 18.7% in 2019 to 97.8% in 2023. This shift signals growing confidence in the technique and reflects the broader trend towards less invasive interventions. Notably, the study demonstrated a 0.9% 30-day mortality rate and a 1.9% rate of major adverse cardiac and cerebrovascular events (MACCEs), reinforcing the notion that transaxillary MICS is not only viable but potentially superior to traditional sternotomy approaches [12].

Several important contributions in this Special Issue further exemplify the advancements and growing scope of MICS: Franz et al. explore the feasibility of minimally invasive mitral valve surgery for patients with infective endocarditis, challenging the notion that this population is unsuitable for less invasive approaches. Salman et al. present hypothermic ventricular fibrillation as a promising strategy for redo mitral valve surgery, addressing the complexities of repeat interventions. Piekarski et al. offer insight into anesthetic protocols critical for ensuring the safety and efficacy of MICS.

Moreover, Taghizadeh-Waghefi et al. focus on the transaxillary approach for high-risk obese patients, demonstrating that MICS can enhance survival and reduce postoperative complications compared to full sternotomy. Ali-Hasan-Al-Saegh et al. likewise delve into the impact of obesity on minimally invasive mitral valve surgery, reinforcing the view that BMI should not limit access to cutting-edge procedures.

Additional articles further enrich the discussion. Moscoso-Ludueña et al. introduce the hybrid concept of combining percutaneous coronary intervention with minimally invasive mitral valve surgery, illustrating the feasibility and safety of this multidisciplinary approach. Meanwhile, Helms et al. emphasize the application of minimally invasive techniques in aortic and hemiarch replacement, underscoring the adaptability of MICS even in complex anatomical scenarios. Kaufeld et al. explore the implications of minimally invasive approaches in thoracic aortic procedures, broadening the scope of this evolving field. Albes et al. provide a comprehensive review on patient selection criteria, offering critical insights into refining procedural indications. Lastly, Weymann et al. present novel data on outcomes following minimally invasive tricuspid valve surgery, further validating the efficacy of MICS across diverse valve pathologies.

Crucially, the success of MICS hinges not only on surgical technique but also on the collaborative efforts of anesthesiologists, perfusionists, and intensive care teams. The Bonn Heart Center protocol for anesthetic management in MICS exemplifies the importance of interdisciplinary coordination in optimizing patient outcomes. Tailored anesthesia protocols, advanced monitoring, and enhanced recovery pathways are essential components that enable the seamless execution of minimally invasive procedures.

The patient-centric benefits of MICS are undeniable. Shorter hospital stays, reduced transfusion requirements, and a faster return to daily activities translate into improved quality of life. A growing body of evidence, including the work by Helms et al. on minimally invasive aortic and hemiarch replacement, suggests that these benefits extend to complex aortic surgeries. This broadens the scope of MICS, reinforcing its role as a cornerstone of modern cardiac surgery.

However, as we embrace the future of MICS, we must also confront the barriers to widespread adoption. The notion that obesity, previous cardiac surgery, or advanced age preclude minimally invasive approaches must be reconsidered. Studies such as those by Ali-Hasan-Al-Saegh et al. demonstrate that obesity is not a contraindication for MICS but rather an opportunity to tailor surgical strategies to individual patient needs. By shifting the focus from patient selection to procedural adaptability, we can expand the reach of MICS and ensure equitable access to its benefits.

In this context, the call to action is clear: “Full sternotomy should soon become an historical access route for valve surgery”. This bold assertion reflects the collective sentiment of this Special Issue’s contributors, who envision a future where minimally invasive techniques are not reserved for select cases but become the standard of care. The emphasis must shift towards selecting the procedure for the patient, not the patient for the procedure.

The future of minimally invasive valve therapies will increasingly depend on specialized centers, as innovation and excellence in these procedures are best cultivated within dedicated institutions.

In this Special Issue, ten accepted papers highlight the forefront of these advancements in minimally invasive cardiac surgery. The contributions are listed below:

▪ Taghizadeh-Waghefi, A.; Petrov, A.; Arzt, S.; Alexiou, K.; Matschke, K.; Kappert, U.; Wilbring, M. Minimally Invasive Aortic Valve Replacement for High-Risk Populations: Transaxillary Access Enhances Survival in Patients with Obesity. *J. Clin. Med.* **2024**, *13*, 6529.
▪ Ali-Hasan-Al-Saegh, S.; Helms, F.; Aburahma, K.; Takemoto, S.; De Manna, N.D.; Amanov, L.; Ius, F.; Karsten, J.; Zubarevich, A.; Schmack, B.; et al. Can obesity serve as a barrier to minimally invasive mitral valve surgery? overcoming the limitations—a multivariate logistic regression analysis. *J. Clin. Med.* **2024**, *13*, 6355.
▪ Salman, J.; Franz, M.; Aburahma, K.; de Manna, N.D.; Tavil, S.; Ali-Hasan-Al-Saegh, S.; Ius, F.; Boethig, D.; Zubarevich, A.; Schmack, B.; et al. Hypothermic ventricular fibrillation in redo minimally invasive mitral valve surgery: a promising solution for a surgical challenge. *J. Clin. Med.* **2024**, *13*, 4269.
▪ Franz, M.; Aburahma, K.; Ius, F.; Ali-Hasan-Al-Saegh, S.; Boethig, D.; Hertel, N.; Zubarevich, A.; Kaufeld, T.; Ruhparwar, A.; Weymann, A.; et al. Minimally invasive surgery through right mini-thoracotomy for mitral valve infective endocarditis: contraindicated or safely possible? *J. Clin. Med.* **2024**, *13*, 4182.
▪ Weymann, A.; Amanov, L.; Beltsios, E.; Arjomandi Rad, A.; Szczechowicz, M.; Merzah, A.S.; Ali-Hasan-Al-Saegh, S.; Schmack, B.; Ismail, I.; Popov, A.-F.; et al. Minimally Invasive Direct Coronary Artery Bypass Grafting: Sixteen Years of Single-Center Experience. *J. Clin. Med.* **2024**, *13*, 3338. https://doi.org/10.3390/jcm13113338.
▪ Helms, F.; Deniz, E.; Krüger, H.; Zubarevich, A.; Schmitto, J.D.; Poyanmehr, R.; Hinteregger, M.; Martens, A.; Weymann, A.; Ruhparwar, A; Schmack, B.; Popov, A.F. Minimally invasive aortic and hemiarch replacement. *J. Clin. Med.* **2024**, *13*, 4406.
▪ Moscoso-Ludueña, M.; Vondran, M.; Irqsusi, M.; Nef, H.; Rastan, A.J.; Ghazy, T. Hybrid approach combining PCI and minimally invasive mitral valve surgery. *J. Clin. Med.* **2024**, *13*, 4406.
▪ Taghizadeh-Waghefi, A.; Petrov, A.; Jatzke, P.; Wilbring, M.; Kappert, U.; Matschke, K.; Alexiou, K.; Arzt, S. Minimally Invasive Isolated Aortic Valve Replacement in a Potential TAVI Cohort of Patients Aged ≥ 75 Years: A Propensity-Matched Analysis. *J. Clin. Med.* **2023**, *12*, 4963. https://doi.org/10.3390/jcm12154963
▪ Claessens, J.; Goris, P.; Yilmaz, A.; Van Genechten, S.; Claes, M.; Packlé, L.; Pierson, M.; Vandenbrande, J.; Kaya, A.; Stessel, B. Patient-Centred Outcomes after Totally Endoscopic Cardiac Surgery: One-Year Follow-Up. *J. Clin. Med.* **2023**, *12*, 4406. https://doi.org/10.3390/jcm12134406.
▪ Piekarski, F.; Rohner, M.; Monsefi, N.; Bakhtiary, F.; Velten, M. Anesthesia for minimal invasive cardiac surgery: the bonn heart center protocol. *J. Clin. Med.* **2024**, *13*, 3939.
